# Childbearing Women’s Experiences of and Interactions With the Health System in Vietnam: A Critical Interpretive Synthesis

**DOI:** 10.1177/2752535X241277678

**Published:** 2024-08-27

**Authors:** Kimberly Lakin, Nguyen Thu Huong, Sumit Kane

**Affiliations:** 1 Nossal Institute for Global Health, Melbourne School of Population and Global Health, 549317 The University of Melbourne, Carlton, VIC, Australia; 2 University of Social Sciences and Humanities, Vietnam National University of Hanoi, Hanoi, Viet Nam

**Keywords:** experiences of healthcare, expectations, maternal health, Vietnam

## Abstract

Scholars have long argued that the care experience is shaped by context, and by evolutions in this context. Using Vietnam as a case, we critically interrogate the literature on women’s experiences with maternity care to unpack whether and if it engages with the major social, economic, and health system impacts of the Doi Moi reforms in Vietnam and with what consequences for equity. We conducted a critical interpretive synthesis of this literature in light of the social, economic, and health system transformations driven by the Doi Moi reforms. We offer three critiques: (1) an overwhelming focus on public maternity care provision in rural/mountainous regions of Vietnam, (2) a narrow focus on women’s ethnic identity, and (3) a misplaced preoccupation with women’s limited autonomy and agency. We argue that future research needs to consider the impact of Vietnam’s shift towards market-oriented care provision, and the broader societal and health system changes impacting both rural and urban areas, as well as ethnic minority and Kinh majority populations.

## Introduction

Recent decades have been characterised by major economic transitions, globally, but particularly in many low- and middle-income countries (LMICs). Economic growth has contributed towards the rapid disappearance of ‘low-income countries’ in almost all regions around the world and, since 1990, the halving of the population living in extreme poverty.^1^ McPake et al.^1^ argue that, as populations become more affluent, there is a growing demand for care that is of a higher-quality, and which meets people’s increasing expectations. In this way, rising living standards, as a consequence of rapid economic growth, can shape how people experience and interact with their health systems. Inquiries examining peoples’ experiences of health and healthcare draw on a long tradition of empirical and theoretical research.^2^ However, historically, both the empirical and theoretical scholarly literature have consistently overlooked the broader social, cultural, historical, and economic systems and structures that can shape how people experience their care.^3^ Particularly, how these systems and structures shape people expectations and experiences of their health system along temporal, spatial, and geographical dimensions.^4,5^ In this paper, we critically interrogate the evidence on women’s experiences with maternity care, within the transitional context of Vietnam, since Doi Moi. We employ a critical interpretive synthesis (CIS) approach to critically unpack to what extent rapid economic, societal, and health system changes, as a result of Doi Moi, are reflected in the current scholarly literature on women’s care encounters. We use the case of women’s experiences with maternity care in Vietnam to illustrate peoples’ experiences with a health system in a country experiencing rapid economic and societal changes and shifts – changes that are akin to those impacting many LMICs.^1^

Vietnam has been dubbed a ‘transition tiger’ – transitioning from a low- to a middle-income economy in a single generation.^6,7^ Such economic growth can be traced right back to Doi Moi – a series of ongoing social and economic reforms, commencing in 1986, that prompted Vietnam’s transition towards a socialist-oriented market economy.^8,9^ These reforms also brought about various health sector developments, central among these, was the liberalisation of the healthcare market with the legalisation of the private sector.^8,9^ In 1993, there were no private health services in the country. However, by 2008, the sector included 83 private hospitals and 30,000 private clinics.^10,11^ As a result, private health services accounted for 40% of total outpatient visits in 2010, and thus play a crucial role in healthcare delivery in Vietnam.^12^ Hospital autonomisation polices have also underpinned the increasing shift of healthcare costs from the State to citizens. As Võ and Löfgren^9^ have revealed, the rise of ‘on-demand’ or ‘patient-requested’ services (such as private service rooms and “high-tech” medical equipment) can strategically serve to maximise the revenue of providers.^9,13^ Therefore, Dao^13^ recently argued that patrial-privatisation, liberalisation, as well as the influx of new medical technologies has changed the organisation of Vietnam’s health system and, with that, peoples’ interactions with the health system. The stratification of Vietnam’s health system, as a result of Doi Moi, has created several distinct, but overlapping “medical routes”.^13^ Primary care is delivered at commune health centres (CHC), with district health centres (DHC) and hospitals, as well as provincial and central hospitals offering more specialised treatment and care. However, recent reviews by the Ministry of Health reveal that many patients bypass CHCs, and even DHCs, in favour of higher-level provincial and central hospitals as they expect better quality of care at these facilities.^14,15^ Currently, there is no guidance to share the examination fees paid by health insurance with CHCs, and the district general hospitals have implemented a self-funded mechanism that leads to high competition to attract patients, resulting in fewer patients visiting the CHCs.^16^ Further competing with public health services are private providers based in private hospitals or outpatient clinics. A national health insurance scheme was launched as part of Doi Moi health sector reforms with benefits for those employed in the formal sector and with low income. However, out-of-pocket payments for care remain high and insurance use is low, compared to the country’s high coverage rate of 87%.^9,13^ This might be attributed to the fact that patients are increasingly seeking care at private facilities and are willing to pay higher fees for perceived better quality of care. In particular, the private sector has been found to better respond to patients’ expectations by having more competent staff, convenient opening hours, and advanced medical equipment.^17^

During pregnancy and childbirth, women and their families are required to navigate these complex “medical routes”, both physically and cognitively.^13^ Examining the pattern of reproductive health service utilisation in Vietnam, Ngo and Hill^17^ found that, while CHCs continued to dominate the provision of antenatal care, urban Kinh women were more likely to seek care at private clinics. Vietnam is an ethnically diverse country with 53 ethnic minority groups co-existing alongside the Kinh majority. Significant gaps in education and socioeconomic conditions however persist between ethnic minorities and the Kinh majority population. Geography – the fact that ethnic minority groups predominantly reside in remote and mountainous regions of Vietnam – only partly explains the difference in living standards between these two groups.^18^ As such, a review of ethnic minority health in Vietnam suggests that ethnic minority women’s preferences for delivering at home are not only driven by distance, but also by cost, cultural rituals associated with childbirth, as well as negative attitudes by health staff. Some scholars have however argued that such explanations are merely rationalisations – distance is relative and there is in fact low knowledge about these culturally-specific practices among health workers.^19^

That said, Ngo and Hill^17^ point to the fact that a significant proportion of women in rural areas, including ethnic minority women, were found to pay the additional costs of visiting private clinics during their last pregnancy. There has yet been no comprehensive synthesis of the literature on women’s experiences of and interactions with maternal health care in Vietnam. Particularly one which critically examines the care encounters of both ethnic minority and Kinh majority women, and in light of the social, economic, and health system transitions initiated by Doi Moi. Currently, the impact of these transitions and changes on women’s engagement with maternity care in Vietnam is insufficiently reflected in much of the empirical literature. Many studies, particularly those conducted with ethnic minority women, seem to predominantly focus on women’s interactions with public health services, despite such facilities failing to meet women’s expectations of short wait times, convenient opening hours, and privacy and confidentiality. Such studies therefore fail to take into account Vietnam’s shift towards market-oriented care provision and the resulting implications for childbearing women’s care-seeking choices and decisions.^20–24^ In light of this, we conducted a critical interpretive synthesis (CIS) to examine whether and if the current literature engages with the major social, economic, and health system impacts of the Doi Moi reforms in Vietnam.

A critical interpretive synthesis (CIS) is an approach to synthesise a diverse body of evidence where the overall aim is interpretive, rather than aggregative.^25^ Several inquiries have used a CIS approach to critically review and synthesise the literature on health systems structures and processes, as well as the experiences and interactions of health system actors, including underserved populations.^26–28^ Our aim of this synthesis was primarily interpretive – we sought to not only present, but critically unpack the current evidence on women’s encounters with maternal health care in Vietnam. To facilitate an interpretive synthesis, the CIS approach emphasises critical appraisal, theory development, and methodological flexibility.^25,29^ It is also guided by a highly iterative compass question, which is constantly refined in response to emerging findings.^25^ The refined compass question which directed our inquiry was: what are childbearing women’s experiences of and interactions with the health system in Vietnam? To respond to this question, we first outline three critiques on how the current empirical literature examines childbearing women’s experiences of their interactions with the Vietnamese health system. These critiques form the basis of our *synthesising argument* which outlines an agenda for future inquires seeking to interrogate childbearing women’s care encounters in contemporary Vietnam.

## Methods

### Literature Search and Selection

A broad search of electronic bibliographic databases was conducted between November 2022 and April 2023. Medline, CINAHL, and PsycInfo databases were searched broadly using the following search terms: (Vietnam OR Viet Nam) AND (maternal* OR maternity OR reproductive* OR perinatal OR peripartum OR antenatal OR prenatal OR intrapartum OR delivery care OR postnatal OR postpartum OR pregnan* OR expectant* OR mother* OR childbirth OR childbearing). The reference lists of review pieces were also hand searched for any relevant literature (including grey literature) which the database search failed to capture.

**Figure 1.**
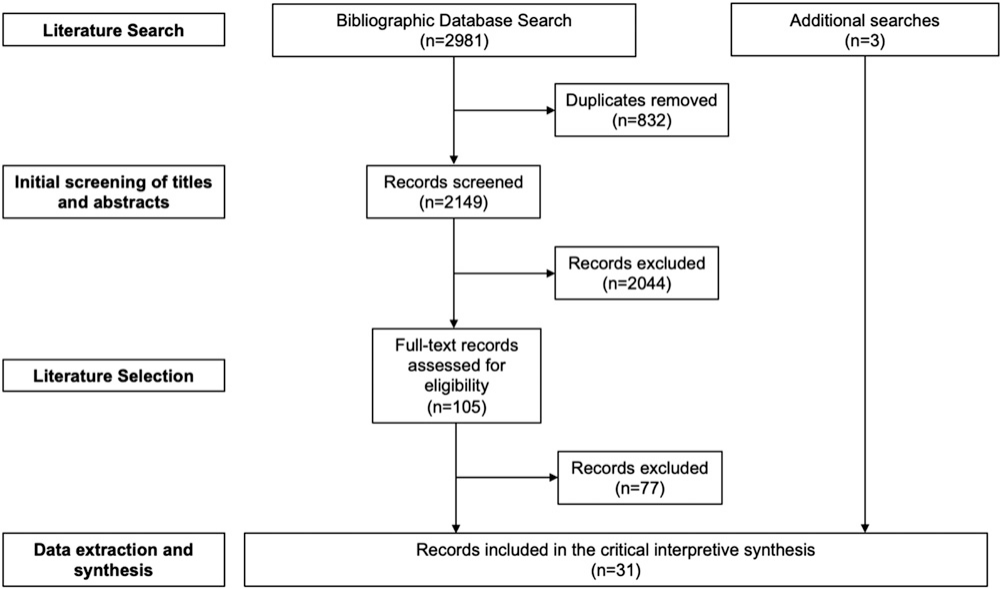
Literature search and selection.

Records captured by the initial database search were imported into an Endnote database. Duplicate records were removed and, as Figure 1 outlines, the titles and abstracts of each article were screened by the first author. Articles which the first author was uncertain about were set aside, and their relevance to the emerging inquiry was discussed with the last author. We broadly included papers which examined women’s experiences of and interactions with maternal health services in Vietnam. As such, we included studies which examined women’s interactions with all levels of maternity care (antenatal, delivery, and postnatal care), at different tiers of the Vietnamese health system (commune, district, provincial, and national), and various health system actors, including both public and private providers. We also included studies which analysed healthcare providers’ and managers’ perspectives on maternal health care provision. However, we excluded studies which did not explicitly examine women’s experiences of their interactions with maternal health care in Vietnam and were thus beyond the scope of this inquiry, including those which related to^1^: breastfeeding practices,^2^ access to and utilisation of abortion services,^3^ Vietnamese migrants’ experiences of maternal health care,^4^ child health and nutrition,^5^ folic acid or iron supplementation during pregnancy,^6^ various health conditions during pregnancy (such as HIV),^7^ adverse pregnancy- and infant-related outcomes (still birth, low birth weight, birth defects), and^8^ family planning services. We also excluded papers which merely measured women’s access to and utilisation of maternal health services without providing an account of their experiences. Moreover, some papers examined women’s experiences of both family planning and maternity care services. These papers were still included, with only data relating to women’s interactions with maternity care analysed. Any uncertainty about the relevancy of papers was resolved by discussion between the authors.

Following initial screening of titles and abstracts, full-text records of included studies were retrieved. These records were then assessed for eligibility and quality by the first author, using Dixon-Woods and colleagues^25^ appraisal prompts.^29^ This involved a two-step process of first considering a paper’s methodological quality (using the five appraisal prompts) and then reflecting on its relevance and theoretical contribution to the emerging inquiry. Only one paper did not adequately report on the methodology for data collection and analysis but, as it offered key insights to the inquiry, it was ultimately included within the CIS. The Preferred Reporting Items for Systematic reviews and Meta-Analyses (PRISMA) checklist guided the reporting of this CIS (see Supplemental file 1).

### Data Extraction and Synthesis

Included papers were imported into QSR NVivo which facilitated data extraction and synthesis. Key characteristics of each paper were first entered into a data extraction proforma, included in Supplemental file 1. Consistent with the CIS approach as proposed by Dixon-Woods et al.,^15^ we adopted an inductive approach to analysis. Each paper was read several times and themes, and sub-themes were coded. For instance, within the theme of “health system”, sub-themes included “accessibility”, “autonomy”, “social support”, and “cost”. Additionally, the theme of “social location” included “ethnicity”, “cultural beliefs”, “disability”, and “rurality” as sub-themes. As Dixon-Woods et al.^25^ propose, our review question served as a “compass rather than an anchor” during this process and was therefore constantly refined in response to the emergent themes. We constantly compared themes and sub-themes against data encountered in the papers to identify notable patterns. This process ultimately facilitated the development of our critiques and overall synthesising argument of the existing scholarly literature on childbearing women’s experiences of and interactions with the Vietnamese health system.

## Results

### Search Results and Article Selection

The electronic bibliographic database search identified a total of 2981 records (see Figure 1). Following the removal of duplicates (*n* = 832), the titles and abstracts of the remaining papers (*n* = 2149) were screened based on the exclusion criteria detailed above. As a result, 105 potentially relevant records were identified and the full-texts of these were examined. A total of 28 records were ultimately included within the CIS. A further three records were identified while reviewing the literature. Details of the included records are presented in Supplemental file 2.

### Childbearing Women’s Experiences of and Interactions with the Health System in Vietnam

In this CIS, we aimed to critically unpack the existing evidence on childbearing women’s experiences of and interactions with the Vietnamese health system. We sought to examine the experiences of both Kinh and ethnic minority childbearing women with different levels of the Vietnamese health system, as well as the perspectives of various health system actors. Yet, we found that the majority of included studies were from sites in rural and mountainous regions of Vietnam i.e., places where the only source of healthcare are the public services. Hence, there is an inevitably disproportionate examination of women’s interactions with public maternity care services which overlooks Vietnam’s growing shift towards market-based provision of healthcare. Because the studies were largely from rural and mountainous regions where ethnic minorities reside, they focused on the care encounters of ethnic minority women. These papers principally focused on the “barriers” preventing such women’s engagement with maternity care and, in this way, primarily problematise women’s “ethnic minority identity”. Finally, much of the literature appears to take a deficit perspective of women’s autonomy and agency during pregnancy and childbirth – overlooking the multiple and complex gender roles, identities, and norms in contemporary Vietnam. Our critiques of the current empirical literature on childbearing women’s encounters with the Vietnamese health system are outlined below.

### Critique 1: a Focus on Maternity Care Provision in Rural/Mountainous Regions of Vietnam

Of the 31 included studies, a total of 20 were conducted in rural/mountainous regions of Vietnam. These inquires principally sought to examine the accessibility and utilisation of maternity care in these areas, drawing on the perspectives of women (including but not limited to ethnic minority women), family members, healthcare professionals (doctors, midwives), managers/directors of local CHCs, as well as other key informants (village health workers, members of the Women’s Union). Across these studies, women residing in rural areas commonly reported a preference for seeking maternity care at CHCs as these primary-level, public facilities were located centrally in each commune.^17,20,21,23,30–32^ Childbearing women described that CHCs in rural areas were deficient in the most basic of amenities – there were few beds for patients, poor sanitation, and even a lack of clean water.^17,20,24^ Such facilities also failed to respond to women’s expectations of convenient opening hours and short waiting times, as well as privacy and confidentiality. Many CHCs did not meet the National Guideline’s recommendation of six separate service rooms – in some cases, there was only one room to accommodate the needs of all patients, including women in labour or those requiring a gynaecological examination.^17,33^ Healthcare providers and managers working at CHCs described how such capacity- and infrastructure-related constraints adversely impacted their ability to respond these needs and demands of childbearing women.^17,33^ Hence, we found that, in these studies, inevitably greater attention was paid towards women’s, healthcare providers’, and managers’ experiences of accessing or providing maternity care specifically at CHCs. That is, primarily examining women’s interactions with public providers.

Yet, we noted that a few studies did describe how the privatisation and commercialisation of Vietnam’s health system, prompted by Doi Moi, has impacted women’s preferences for, and subsequent interactions with private providers – even in rural areas. For instance, Gammeltoft^34^ suggested that “high-tech” medical equipment (such as obstetric ultrasounds) can serve as “revenue-generating services” for both public and private providers to “attract more patient”.^17,35,36^ As a result, and as highlighted in a few studies, this has potentially contributed towards the overuse and commercialisation of obstetric ultrasounds in Vietnam. However, as Ngo and Hill^17^ reveal, these revenue-maximising practices were rarely acknowledged by providers, though they recognised that women’s increasing use of ultrasounds was a sign that, *“…today, our lives have improved, social conditions have improved, and so people care more about their health. They also have more knowledge, culturally we have advanced”*.

Private clinics, in particular, were perceived to respond to women’s demands for high-quality ultrasounds and some studies revealed how this prompted women’s interactions with private providers.^20,34–37^ For instance, in Heo et al.’s^38^ study, women preferred visiting private providers for antenatal care due to flexible opening hours and access to high-quality, colour ultrasounds. Women reported that they were willing to pay for visiting private providers, and for colour ultrasonography, as they felt the cost was affordable.^38^ Heo et al.’s^38^ study was situated in a rural district in Vietnam, but one that was also experiencing rapid urbanisation. Thus, it potentially serves as an illustrative example of how Doi Moi-related reforms are also gradually impinging upon the health system in rural regions of country.^34,35,37,39^ In fact, a study by McKinn et al.^20^ revealed how even childbearing ethnic minority women residing in a rural district perceived the availability of ultrasounds as an incentive for visiting a private clinic – ultrasounds were not offered at CHCs. Given this, we call for a deeper engagement with and critical analysis of how Vietnam’s shift towards market-oriented provision of healthcare has impacted childbearing women’s care encounters. This particularly includes inquiries set in rural/mountainous regions of Vietnam which, as the aforementioned studies suggest, are not altogether untouched by market forces triggered by Doi Moi.

### Critique 2: the Problematisation of Ethnicity

Many studies specifically examined the experiences of childbearing ethnic minority women’s encounters with the health system. Within these inquiries, there was an inordinate focus on the “barriers” that prevented ethnic minority women from accessing antenatal or delivery care from a health facility. Some ethnic minority communities, such as the Hmong, lived far from the public health facilities.^23^ The distance, coupled with expensive transportation and poor road conditions, were found to deter ethnic minority women from accessing care, particularly when giving birth.^19,40,41^ If ethnic minority women did seek care, mostly at a local CHC, the facility was found to be poorly constructed and lacked essential resources, such as beds (described further above).^20^ Some women reported experiencing negative attitudes from health staff and were unable to speak or understand the Kinh language – the official language of Vietnam.^19,33^

Also presented as “barriers” in these inquires, were “vaguely-defined” traditional customs associated with pregnancy and birth.^21^ For instance, in the Hmong ethnic minority community, the placenta can be seen as a “crucial symbol of the cycle of mortal life” and is ritualistically buried following birth.^40,42^ However, in Corbett et al.’s^40^ study, giving birth in a hospital was found to potentially “disrupt” this traditional ritual. The lack of cultural sensitivity and language barriers among healthcare workers working within ethnic minority communities can lead to poor communication regarding health issues and further discourage care-seeking among ethnic minority women.^21^ More generally, some studies associated ethnic minority women’s “shyness” with a hesitancy to seek care from a facility or to ask healthcare providers questions.^33,43^ Yet, White et al.^21^ found that the speed or ease of delivery was the most dominant explanation women provided for giving birth at home – “shyness” was rarely cited as an explanation. This study reveals that ethnic minority women continued to practice sitting, squatting, and kneeling delivery positions, and discussed the importance of the presence of husbands and other support persons during home childbirth. The National Standard Guidelines on Reproductive Health however does not take into account any of these practices and preferences.^44^

Within these inquiries, the overwhelming emphasis on the “barriers” that impede ethnic minority women’s access to and utilisation of maternity care services, particularly those related to traditional customs or rituals, “implicitly place the onus of minority women to change behaviour and engage more comprehensively with services”.^21^ This problematic presentation was in fact reflected within a sub-section of a UNFPA^42^ report which detailed, *“the problem of ethnic minority groups and low usage of public birth facilities”*. In this way, we argue that such inquires “problematise” women’s ethnic minority identity. That is, present women’s ethnic minority status as a “problem” or “barrier” to accessing and utilising maternity care services. There were even instances where health professionals were found to “blame” ethnic minority women who were referred to higher-level facilities for care: *“ethnic people from mountainous and remote areas come here, and it is a big problem because the total number of daily patients increases”*.^45^ Several studies engaged with this problematic presentation of childbearing ethnic minority women as “obstacles” to service attendance which, as White et al.^21^ argues, can be seen as conforming to mainstream stereotypes in Vietnam which enforce the “otherness” or “backwardness” of ethnic minorities. Such inordinate problematisation of childbearing women’s “ethnic minority identity” in the literature, we contend, can shift the focus away from improving overall service capacity and quality. Particularly, as both Kinh and ethnic minority women alike could be the recipients of poor-quality care in rural/remote regions of Vietnam (as we have highlighted above).^45^ This was emphasised in Binder-Finnema et al.’s^45^ study in which a health professional suggested that *“… there is no difference in wanting to provide healthcare to Kinh people or Thai people”*.

Moreover, the narrow focus on “ethnicity”, we argue, overlooks how other social systems and structures can shape childbearing women’s care encounters, including but not limited to, the encounters of Kinh women. A few studies analysed the care experiences of women with physical disabilities in both urban and rural Vietnam.^39,46–49^ Such women faced numerous accessibility-related issues; toilets were not accessible for wheelchair use and there was an absence of ramps and lifts. As such, health facilities, particularly public hospitals, failed to meet women’s needs and, in some instances, they experienced undignified treatment by health staff.^47,49^ Another study, by Klingberg-Allvin et al.,^50^ unpacked the experiences of adolescent mothers, finding that such women “normalised” patronising and discriminative treatment by healthcare staff. We argue that the dominant focus on women’s “ethnic minority identity” and the “obstacles” it elicits, not only conforms and entrenches prevailing stereotypes, but also overlooks the experiences of childbearing women with different lived experiences.

### Critique 3: Childbearing Women’s Limited Autonomy and Agency

Finally, throughout much of the literature, there is an emphasis on women’s unequal decision-making power during pregnancy and childbirth. The Confucian belief-system foregrounded numerous inquiries, which is said to define the patriarchal Vietnamese family structure.^24,32,51^ As such, the husband (and his family) may have a particular influence on women’s decision-making relating their sexual and reproductive health. In several inquiries, influenced by Confucian patrilineal practices, women were pressured by their husbands and parents-in-law to have son.^20,24^ This was exemplified in the study by McKinn et al.,^20^ where a woman indicated that she would continue to try and have a son to fulfill her husband’s wishes. This was against the advice of health professionals and prompted her to travel far to the provincial capital to determine the sex of her baby via ultrasound. The unequal decision-making power of women was even evident when it came to the choice of antenatal or delivery facility. In Duong et al.’s^30^ study, a woman admitted, *“his mother decided the whole thing”* and, McKinn et al.^20^ reported how an ethnic minority woman’s parents advised her against visiting a CHC as they felt that one antenatal check-up was “enough”. As such, some studies highlighted how women’s limited decision-making power was particularly prevalent in ethnic minority communities where men, fathers, and patriarchs can be viewed as the ‘gatekeepers’ in prompting access to reproductive health services.^20,52^ When it came to the care encounter itself, several studies revealed how women had limited autonomy when consulting with health professionals.^20,36,46,47^ Traditional gender roles and the high regard for the medical professional could indeed explain why women followed the advice of doctors without question. Yet, one study did point out that women can be torn between the advice of health professionals and family members.^46^ However, when deciding where to seek care, the preferences of family members seemed to prevail and, in some cases, the decision was taken out of women’s hands entirely.^20^ Overall, much of the examined literature appears to take a deficit perspective of childbearing women’s autonomy during pregnancy and childbirth and, in this way, potentially presents Vietnamese women as “powerless”.

However, there were exceptions. As opposed to the findings above, Heo et al.’s^38^ study revealed that women encountered few cultural and social barriers to accessing maternal and child health services. Women were reported to have a “high level to autonomy” and were able to make their own decision on where to seek care. These decisions were informed by drawing on formal and informal sources of information, such as the internet and older, experienced women who were seen as trusted sources of knowledge.^20,38^ However, some women conceded that the knowledge of other women was based on experience, while the advice offered by health professionals was science-based and, as a result, more reliable.^32^ These examples suggest that childbearing women in Vietnam may not in fact be passive recipients of healthcare, as the evidence above suggests; they appear to exercise their agency and autonomy in a variety of ways, particularly by drawing on the expertise of others to make an informed choice on where to seek care. Another source of knowledge may also be a woman’s husband and a few studies revealed instances of joint decision-making when it came to the birth. For instances, in the study by White et al.,^21^ a Thai ethnic minority woman reflected, *“I discussed things with my husband, and we decided that if the delivery was easy, I would deliver at home. If the labour was difficult, I would go to the CHC.”*. However, as Duong et al.^30^ found, joint decision-making may suggest that traditional gender roles and structures still pose an obstacle to childbearing women’s autonomy. While women were empowered to manage the family’s finances, the ultimate decision on spending still resided with the husband. In this way, women were reluctant to independently make the final decision on where to seek care.^30^ These examples hint at the existence of multiple and complex gender roles, identities, and norms in contemporary Vietnam.^53^ However, this nuance, we argue, is overlooked by inquires which adopt a deficit perspective by solely examining the impact of patriarchal norms and conventions on childbearing women’s autonomy and agency during pregnancy and childbirth.

## Discussion

In the three critiques outlined above, we aimed to critically unpack how the current literature examines childbearing women’s experiences of and interactions with the health system in Vietnam. Drawing together the concepts emerging within these critiques, we propose the following overarching *synthesising argument*:

Vietnam’s shift towards market-oriented provision of healthcare is insufficiently reflected in much of the current literature, which is primarily set in rural/mountainous regions of the country and examines women’s encounters with public maternity care services. Moreover, the resulting dominant focus on the experiences of ethnic minority women not only inordinately problematises the ‘ethnic minority identity’ but obscures the experiences of the vast majority of Kinh women and the impact of other social structures and systems on their lives and care-seeking behaviours. Finally, the presence of multiple, complex, and competing gender roles, identities, and norms, we suggest, is overlooked by studies which emphasise childbearing Vietnamese women’s limited autonomy and agency. We argue that future inquires seeking to interrogate childbearing Vietnamese women’s care encounters should explicitly take into account the social, economic, and health system transitions that currently define contemporary Vietnam.

Below we discuss the implications of these critiques and our findings for future research, policy, and practice in Vietnam.

### Recognising Vietnam’s Shift towards Market-Oriented Provision of Care

The Doi Moi reforms of the 1980s saw an increase in private and foreign investment which led to rapid economic growth, particularly in major cities, and encouraged rural-urban migration. These economic reforms have transformed Vietnam into a middle-income economy and has seen rates of absolute poverty rapidly fall.^8^ Within the health system, the reforms saw the exponential growth of the private healthcare sector and the “covert” privatisation of the public health system. In this way, health system changes prompted by Doi Moi have increased both the heath care options available to service users, as well as the cost of care. As a result, and as Dao^13^ recently proposed, several new “medical routes” have emerged. That said, in the vast majority of included studies, which were set in rural/mountainous regions of Vietnam, there was inevitably a greater focus on childbearing Vietnamese women’s (including ethnic minority women) experiences related to the “public” medical route. In these studies, women primarily sought care at local CHCs, despite such facilities failing to respond to their expectations of convenient opening hours and short waiting times, as well as privacy and confidentiality.

Yet, few studies did shed light on how the growing privatisation and commercialisation of Vietnam’s health system prompted childbearing women to alternatively embark on a “private” medical route, even for those residing in a rural district. In these studies, private clinics were found to be more responsive to women’s needs and demands, especially with regards to having access to high-quality ultrasounds.^20,34–38^ McPake et al.^1^ have argued that, as people become more affluent, as a result of rapid economic growth, they expect care that is of a greater quality and cost. Indeed, Heo et al.’s^38^ study showed how women were willing to pay the costs of visiting a private provider to meet their expectations of timeliness and high-quality ultrasounds. Even in this rural district, women felt such costs were affordable. In the study by Ngo and Hill,^17^ a health professional suggested that women’s ability to access such advanced medical technologies is a sign that “social conditions have improved” and that Vietnam has advanced both culturally and in terms of the population’s health and wellbeing. As evidence in Critique 1 suggests, Doi Moi-related reforms and its impact are not exclusively restricted to the health system in urban/suburban areas, nor to the care encounters of Kinh majority women. We argue that any future inquiry (regardless of setting or population group) seeking to examine the care experiences of childbearing Vietnamese women should consciously and explicitly consider the impact of Doi Moi on the organisation, practices, structures, norms, and activities of Vietnam’s health system. But also, how such health system reforms and changes shape what childbearing women expect from the health system and their subsequent decisions, choices, preferences, and interactions with health services.

### Moving Beyond a Narrow and Sole Focus on “Ethnic Minority Identity”

As discussed above and in Critique 2, the vast majority of literature examined childbearing women’s, and especially ethnic minority women’s, preferences for and engagements with public health services. For ethnic minority women, this could be due to the fact that they, as well as those from ‘poor’ households, are entitled for free check-ups and (basic) medicines at CHCs. In some cases, when there may be no early signs of pregnancy-related complications, reluctance to use the government healthcare services among some ethnic minority communities may be related to a strategy of “selective non-participation”. The history of mistreatment by, and resulting mistrust towards, national governments may explain this, and such a finding aligns broadly with what has been called “the art of not being governed”.^23^ Yet, there was evidence that, even in ethnic minority communities, ultrasounds were an incentive for women to visit private providers and generally served as a maker of high-quality care.^20,34–37^ Nevertheless, within many inquiries, there was an overwhelming emphasis on the “barriers” that women’s “ethnic minority identity” presents to accessing and utilising maternity care services.

We argue that any examination of how childbearing women’s “ethnic minority identity” shapes their experiences of and interactions with the health system, should explicitly consider the diversity and heterogeneity of ethnic minority groups in Vietnam. The narrow and sole focus on women’s “ethnic minority identity”, we contend, can reinforce and propagate prevailing stereotypes, while also potentially presenting all 53 ethnic minority communities as homogenous. This problematic presentation can ignore inequalities and disparities between and within ethnic minority groups too. For instance, the recent study by the World Bank^54^ revealed that relative to other minorities, utilisation of maternal health services is particularly low among the Hmông, Khơ Mú, and Xơ Đăng. A ‘one-size-fits-all’ approach to ethnic minority health and wellbeing in Vietnam^21^ is thus clearly problematic at many levels. Work by Liu et al.^55^ has highlighted the importance of viewing ethnicity as dynamic, intersecting with age, gender, and other social structures to shape lived experiences. An intersectionality-informed approach, which considers ethnicity as it intersects with other social structures, we argue, can allow researchers, practitioners, and policymakers to move beyond the narrow and one ‘one-size-fits-all’ approach to examining and improving ethnic minority health in Vietnam and globally.^19^

### Considering the Multiple and Complex Gender Roles, Identities, and Norms in Contemporary Vietnam

The gendered impacts of Doi Moi prompted Werner^56^ to argue that the economic restructuring of Doi Moi is not merely a series of economic reform policies but a “socially embedded process” that is shaped by many gendered components. One such impact of the economic reforms is that relations and workloads between men and women, both within the “public” and “private” spheres, are increasingly unequal; women not only have to maintain a “socialist work ethic” but must also attend to household duties.^56,57^ These gender dynamics bear upon all aspects of social life, particularly, as we have revealed above, the household dynamics of seeking care. However, in Critique 3, we have pointed out how much of the examined literature referred to Confucian patrilineal practices and norms to highlight the unequal decision-making power and agency of childbearing women in Vietnam – women’s decision on seeking care during pregnancy and childbirth is heavily influenced by the preferences of her husband and/or in-laws.^20,24,30^

However, other studies revealed that childbearing Vietnamese women are not simply “passive agents” in seeking care; they exhibited a “high degree of autonomy”, encountering few cultural and social barriers to accessing maternal health services.^20,24,38^ We contend that this evidence points to the existence of diverse, multiple, and sometimes contradictory gender roles, identities, and norms in Vietnam. For instance, echoing work by Long et al.,^57^ Duong et al.’s^30^ study found that, women appear to have greater control over financial resources within the household. Similarly, work by Do and Brennan^58^ has highlighted the symbolic and practical power that women have over men within the ‘domestic sphere’, particularly in relation to the family economy, which they suggest has created even more complexity in the dynamics of power relations in contemporary Vietnam.

Yet, as Duong et al.’s^30^ study found, women can still be required to consult their husband before spending money and may be reluctant to make the final decision on where to seek care. Moreover, Do and Brennan^58^ emphasise that power, as a dimension of gender, is often associated with patriarchy and ‘formal power’. Their study, and the findings we outline in Critique 3, shed light on the idea of ‘power outside the symbols of power’. Several studies showed how women drew on their ‘informal power’ to consult with formal and informal sources of information, such as the internet and older, experienced women to decide where to seek care.^20,24,33^ We argue, as do Long et al.,^57^ that a focus on Confucian and/or patriarchal traditions alone does not sufficiently explain the complex and competing gender roles, identities, and norms in contemporary Vietnam, which have arisen from the shift towards a market economy. We therefore call for future research to move beyond adopting a deficit approach to examining childbearing women’s care-seeking in Vietnam. Instead, there is a need for researchers to consider what Do and Brennan^58^ have called, the “paradox of power” that Vietnamese women encounter during their pregnancy and childbirth in both public and private spheres. This would require further research to examine how the Doi Moi-driven social and economic changes have shaped complex gender dynamics and power relations in Vietnam today, in both rural and urban regions of the country, and for ethnic minority and Kinh women alike.

## Limitations

Critical interpretive syntheses prioritise theoretical relevance over methodological quality. As such, we acknowledge that a potential limitation of such an approach is the inclusion of methodologically weak papers. While critically appraising the literature, we did carefully examine the methodological quality of each paper and identified that one paper did not adequately report on the methodology for data collection and analysis. We have however been transparent about including this paper in the CIS as it offered key insights to the inquiry. Another limitation of conducting a CIS, as Depraetere and colleagues^29^ suggest, is that there can be inter-study variability with respect to the processes and reporting standards of CIS. In recognition of this limitation, we have transparently reported the processes of literature selection and critical appraisal we followed (in line with the adapted PRISMA checklist), acknowledging the prioritisation of theoretical contribution of studies over methodological quality.

## Conclusion

Major economic transitions have impacted many LMICs in recent decades and have shaped how people experience and interact with their health systems. Historically, however, both the empirical and theoretical scholarly literature have consistently overlooked the broader social, cultural, and economic systems that can shape how people experience their care. Using maternity-related care seeking in Vietnam as an illustrative case, we conducted a critical interpretive synthesis of this literature, taking into account the social, economic, and health system transformations driven by the Doi Moi reforms. We demonstrate how the current literature disproportionately examines women’s interactions with public maternity care services in rural/mountainous regions of the country, problematises women’s “ethnic minority” identity, and emphasises childbearing women’s limited autonomy and agency. Such findings call to attention the need for future research to engage with the country’s shift towards market-oriented provision of care, move beyond a narrow focus on “ethnic minority” identity, and consider the complex gender dynamics that currently define contemporary Vietnam.

## Supplemental Material

Supplemental Material - Childbearing Women’s Experiences of and Interactions With the Health System in Vietnam: A Critical Interpretive Synthesis Supplemental Material for Childbearing Women’s Experiences of and Interactions With the Health System in Vietnam: A Critical Interpretive Synthesis by Kimberly Lakin, Nguyen Thu Huong, and Sumit Kane in Community Health Equity Research & Policy.

Supplemental Material - Childbearing Women’s Experiences of and Interactions With the Health System in Vietnam: A Critical Interpretive Synthesis Supplemental Material for Childbearing Women’s Experiences of and Interactions With the Health System in Vietnam: A Critical Interpretive Synthesis by Kimberly Lakin, Nguyen Thu Huong, and Sumit Kane in Community Health Equity Research & Policy.
