# Supplementary material for: Childbearing Women’s Experiences of and Interactions With the Health System in Vietnam: A Critical Interpretive Synthesis
Source: Community Health Equity Res Policy. 2024 Aug 27;45(4):447–57. doi: 10.1177/2752535X241277678 (PMC12059237; doi:10.1177/2752535X241277678)
Supplement: Supplemental Material - Childbearing Women’s Experiences of and Interactions With the Health System in Vietnam: A Critical Interpretive Synthesis [file sj-pdf-2-qch-10.1177_2752535X241277678.pdf]

Supplementary file 2: Data extraction proforma

Record	Publication year	Aims/objectives	Type of source	Methodology	Participant characteristics	Setting (rural, semi-urban, urban)	Level of the health system examined (private, commune, district, provincial, national/central)
Binder-Finnema, P., Lien, P. T., Hoa, D. T., & Malqvist, M.	2015	Identify underlying structural barriers to equitable maternal health care in Nghe An province, Vietnam.	Research article	Qualitative (focus group discussions)	1) Kinh, Thai and Tho ethnic minority mothers,<br>2) Maternal healthcare providers	Rural Nghe An province (Kinh or ethnic minority communities)	Various levels of maternity care
Corbett, C. A., Callister, L. C., Peterson Gettys, J., & Hickman, J. R.	2017	To provide an ethnographic view of the perspectives on birthing of Hmong mothers living in the highlands of Vietnam.	Research article	Ethnography	Hmong women (given birth in the last 17 months)	Rural Hmong villages in Sapa	Various levels of maternity care
Duong, D. V., Binns, C. W., & Lee, A. H.	2004	To investigate factors that influence the utilization of delivery services at the primary health care level in rural Vietnam	Research article	Mixed-methods (survey, focus groups and in-depth interviews)	Women who have recently given birth	Rural Quang Xuong District, Thanh Hoa Province	Commune-level
Duong Thi Thuy, D., Tolib, M., Canh Chuong, N., & Ha Thi Thu, B.	2018	Reports findings of implementation research that aimed to increase the acceptability of village-based ethnic minority midwives (EMMs) by local communities in Vietnam.	Research article	Mixed methods (questionnaire, in-depth interviews and focus groups)	1) Ethnic minority midwives,<br>2) Relatives of pregnant women,<br>3) Community representatives, and<br>4) health managers	Rural, highland regions of Dien Bien and Kon Tum	Community-level
Edvardsson, K., Graner, S., Thi, L. P., Åhman, A., Small, R., Lalos, A., & Mogren, I.	2015	To explore Vietnamese obstetricians' experiences and views on the role of obstetric ultrasound in relation to clinical management of complicated pregnancy.	Research article	Qualitative (in-depth interviews)	Obstetricians and sonographers	Rural, urban, and suburban regions of Northern Vietnam	Various levels of maternity care
Gammeltoft, T., & Nguyen, H. T.	2007	Examine the use of obstetric ultrasonography in routine antenatal care in Hanoi, Vietnam.	Research article	Mixed-methods (observations, survey and individual interviews)	1) Pregnant women<br>2) Doctors providing ultrasounds<br>3) Senior doctors and<br>4) Ministry of Health officials	Hanoi	National level
Gammeltoft, T.	2007	To show how Hanoian women's paradoxical stances toward ultrasound imaging can	Research article	Ethnography	Mothers of young children	Inner and outer districts of Hanoi	Various levels of maternity care

		be explained through a consideration of embodied and historically generated experiences within everyday local worlds.					
Graner, S., Mogren, I., Duong le, Q., Krantz, G., & Klingberg-Allvin, M.	2010	Explore the perspectives and experiences of midwives, assistant physicians and medical doctors on the content and quality of maternal health care in rural Vietnam.	Research article	Qualitative (focus group discussions)	Midwives,<br>Medical doctors and Assistant physicians	Rural Bavi district	Commune and district level
Graner, S., Klingberg-Allvin, M., Duong le, Q., Krantz, G., & Mogren, I.	2013	Explore perceptions and experiences among pregnant women in rural Vietnam with respect to pregnancy-related risks and maternal health care.	Research article	Qualitative (focus group discussions)	Kinh and Muong ethnic minority pregnant women	Rural Bavi district	Commune and district level
Ha, B. T. T., Huong, N. T. T., & Duong, D. T. T.	2017	To provide information on the prenatal diagnostic (PND) services provided in three major regional PND centers in Vietnam.	Research article	Cross-sectional (document review and in-depth interviews)	Various key informants (hospital manager, physician, technician and midwife)	Hospitals in Hanoi, Hue city and Ho Chi Minh City	National level
Heo, J., Kim, S. Y., Yi, J., Yu, S. Y., Jung, D. E., Lee, S., Jung, J. Y., Kim, H., Do, N., Lee, H. Y., Nam, Y. S., Hoang, V. M., Luu, N. H., Lee, J. K., Tran, T. G. H., & Oh, J.	2020	Examine current maternal, newborn and child health service utilization patterns at a district level.	Research article	Qualitative (in-depth interviews and focus group discussions)	1) Women who recently gave birth<br>2) Healthcare professionals and managers/directors of CHS	Rural district of Quc Oai (now experiencing rapid urbanisation)	Commune and district level
Holmlund, S., Lan, P. T., Edvardsson, K., Ntaganira, J., Graner, S., Small, R., & Mogren, I.	2020	To explore Vietnamese midwives' experiences and views on the role of obstetric ultrasound in relation to clinical management, including ethical aspects.	Research article	Qualitative (focus group discussions)	Midwives working at district and national level hospitals	Urban, semi-urban and rural parts of Hanoi	District to national levels
Holmlund, S., Lan, P. T., Edvardsson, K., Ntaganira, J., Graner, S., Small, R., & Mogren, I.	2022	To explore Vietnamese midwives' experiences of working in maternity care.	Research article	Qualitative (focus group discussions)	Midwives working in hospitals within district, provincial and national level hospitals	Urban, semi-urban and rural parts of Hanoi	District, provincial and national levels
Huong, N. T. T., Anh, H. P., Hao, M. T. T., & Huyen, N. T. H.	2021	To examine the current knowledge, attitudes, and practice of parents about maternal care in a mountainous region of Cao Bang province.	Research article	Qualitative (in-depth interviews and focus group discussions)	Both Kinh and Mong ethnic minority parents (including pregnant women or mothers)	Mountainous region of Cao Bang province	Various levels of maternity care
Klingberg-Allvin, M., Binh, N., Johansson, A., & Berggren, V.	2008	Explores married Vietnamese adolescents' perceptions and experiences related to transition into motherhood and	Research article	Qualitative (in-depth interviews)	Women younger than 20 who were either pregnant or had newly delivered.	Rural district north of Hanoi	Various levels of maternity care

		their encounter with health care services.					
McBride, B., O'Neil, J. D., Hue, T. T., Eni, R., Nguyen, C. V., & Nguyen, L. T.	2018	To evaluate a low-cost mobile health (mHealth) intervention to improve access to maternal, newborn and child health (MNCH) services and health equity among EMW living in remote areas.	Research article	Qualitative (in-depth interviews)	1) Ethnic minority women,<br>2) Husbands,<br>3) Healthcare workers, and<br>4) Mangers	Thai Nguyen (mountainous) province	Various levels of maternity care
McKinn, S., Linh Thuy, D., Foster, K., & McCaffery, K.	2017	To explore how ethnic minority women experience communication with primary care health professionals in the maternal and child health setting.	Research article	Qualitative focused ethnography (focus group discussions)	Women who were currently pregnant, or who had been pregnant in the previous five years	Rural Tuan Giao district in Dien Bien Province	Various levels of maternity care
McKinn, S., Linh, D. T., Foster, K., & McCaffery, K.	2019	To explore the nature of maternal health literacy among ethnic minority women in a low-resource setting in Vietnam	Research article	Qualitative (focus group discussions)	1) Thai and Hmong ethnic minority women (currently pregnant, mothers or grandmothers)<br>2) Key informants	Rural Tuan Giao district in Dien Bien province (large ethnic minority population)	Various levels of maternity care
McKinn, S., Linh, D. T., Foster, K., & McCaffery, K	2019	1) How and why ethnic minority women currently use and do not use maternal healthcare services,<br>2) Identify the factors that influence ethnic minority women and their families in their decisions to seek maternal healthcare services, and the barriers and facilitators to preventive care seeking	Research article	Qualitative (in-depth interviews)	1) Health professionals from CHS<br>2) Thai and Hmong ethnic minority women (currently pregnant, mothers or grandmothers)<br>3) Village health worker and village midwife	Rural district in Dien Bien province (high ethnic minority population)	Commune and district level
Ngo, A. D., & Hill, P. S.	2011	To explore the challenges faced by local CHSs in three provinces of Vietnam and the extent to which they had achieved universal access to community-based primary reproductive health care in the context of an increasingly commercialised health market.	Research article	Qualitative (in-depth interviews and focus group discussions)	1) Heads of CHS<br>2) Midwives<br>3) Women patients	Thai Nguyen (mountainous), Thua Thien Hue (urban, rural and mountainous areas) and Vinh Long (rural)	Commune level
Nguyen Thi Hoai, T., Wilson, A., McDonald, F., & Thu, N. T. H.	2015	To examine the impact of motivation on maintenance of professional competence among maternal health workers in Vietnam.	Research article	Mixed-methods (survey and in-depth interviews)	Maternal healthcare workers from CHCs and district hospitals	Five rural districts of two northern mountainous provinces of Vietnam	Commune and district level
Nguyen, A.,	2019	To explore the reproductive	Research article	Qualitative (in-depth	Men and women with	Ho Chi Minh City	Various levels of

Liamputtong, P., & Horey, D.		health care experiences of people with physical disabilities in Vietnam.		interviews with the use of drawing and photo elicitation methods)	physical disabilities		reproductive care (only data relating to maternity care was coded)
Nguyen, T. V., King, J., Edwards, N., & Dunne, M. P.	2021	To explore the lived experiences of women with physical disabilities through their pregnancy journeys, using an intersectional lens.	Research article	Qualitative (in-depth interviews)	Women with physical disabilities who had given birth in the previous three years	Northern provinces of Hanoi and Thai Binh	Various levels of maternity care
Nguyen, T. V., King, J., Edwards, N., & Dunne, M. P.	2022	To explore the relative influence of health sta, family, friends, and the women themselves on key decisions about childbirth of women with physical disabilities in northern Vietnam.	Research article	Qualitative (in-depth interviews)	1) Women with physical disabilities who had given birth in the previous three years<br>2) Maternal healthcare providers	Northern provinces of Hanoi (urban) and Thai Binh (rural)	Various levels of maternity care
Nguyen, T. V., King, J., Edwards, N., & Dunne, M. P.	2022	To explore how maternal healthcare access was experienced by women with physical disabilities in Northern Vietnam.	Research article	Qualitative (in-depth interviews)	Women with physical disabilities who recently gave birth	Hanoi city (urban) and Thai Binh province (rural)	Various levels of maternity care
Nguyen, T. V., Edwards, N., & King, J.	2023	Explores perspectives and experiences of maternal healthcare providers in the delivery of services to women with physical disabilities in Northern Vietnam.	Research article	Qualitative (in-depth interviews)	Healthcare providers working in both public and private facilities	Hanoi city (urban) and Thai Binh province (rural)	Various levels of maternity care
Thi Hoai Thu, N., McDonald, F., Witter, S., & Wilson, A.	2018	To explore how district level reforms impact the organisation of maternal health care delivery at district and commune levels.	Research article	Qualitative (in-depth interviews)	Health staff and managers involved in the provision of maternal health services from commune to the central levels	Rural districts in Bac Giang and Lao Cai	Commune and district levels
Van, T. V., Ngoc, H. L., & van Schie, T. J.	2004	To determine the barriers to the use of maternal care and family planning (services by the disadvantaged Kinh people and Katu ethnic minority people	Research article	Mixed methods (survey, in-depth interviews and focus groups)	Mothers with at least one child under the age of 5 year<br>Health staff,<br>Managers	Remote and mountainous area of Nam Dong District in Central Vietnam	Various levels of family planning and maternity care (only data relating to maternity care was coded)
White, J., Oosterhoff, P., & Huong, N. T.	2012	To enhance understanding of childbirth practices and maternal health-seeking behaviour among local Thai and Hmong villagers.	Research article	Qualitative (secondary data from literature review and primary data from focus group discussions)	Thai and Hmong ethnic minority women	Ha Giang and Dien Bien provinces (one remote Hmong village, one Thai village and one Kinh village)	Various levels of maternity care
United Nation Population Fund (UNFPA).	2007	To document reproductive health knowledge and behaviour of the ethnic minority community in	Research article	Qualitative (in-depth interviews and focus groups)	1) Reproductive healthcare providers<br>2) Clients	Rural provinces of Hoa Binh and Ha Giang	Various levels of reproductive care (only data relating to maternity care was coded)

		mountainous provinces			3) Mass community organisations (Women's Union, Youth's Union)		
United Nation Population Fund (UNFPA).	2008	To explore reproductive health services provided to ethnic minority people living in three communes located in mountainous and remote districts of Binh Dinh province	Research article	Qualitative (in-depth interviews, focus groups, observations)	1) Ethnic minority women in the reproductive age group 15-45<br>2) Providers of reproductive health care	Three communes in rural districts of Binh Dinh province	Various levels of reproductive care (only data relating to maternity care was coded)
